# The complete genome sequence of Neckar River virus confirms it to be a distinct member of the genus *Tombusvirus* in the family *Tombusviridae*

**DOI:** 10.1007/s00705-023-05918-z

**Published:** 2023-11-20

**Authors:** Thi Chi Tran, Edgar Maiss, Hanna Rose

**Affiliations:** Department of Phytomedicine, Institute of Horticultural Production Systems, Leibniz University Hannover, Herrenhäuser Str. 2, 30419 Hannover, Germany

## Abstract

Neckar River virus (NRV), first isolated from a water sample of the Neckar River (Germany) in the 1980s, was serologically characterized as a novel tombusvirus. In this study, the complete genome sequence was determined, and an infectious full-length cDNA clone was constructed. The genome organization of NRV (DSMZ PV-0270) resembles that of tombusviruses. The genome consists of 4739 nucleotides and contains five open reading frames (ORFs) and one additional putative ORF (pX) in the 3’-terminal region. Phylogenetic analysis and sequence comparisons confirmed NRV to be a member of the species *Tombusvirus neckarfluminis* in the genus *Tombusvirus*. The infectious full-length cDNA clone was constructed using Gibson assembly and subsequent infection of *Nicotiana benthamiana* plants by *Rhizobium radiobacter* inoculation. The virus derived from the full-length cDNA clone caused symptoms resembling those caused by the wild-type virus, but slightly milder.

**Supplementary Information:**

The online version contains supplementary material available at 10.1007/s00705-023-05918-z.

Tombusviruses from surface water of German rivers were first described in the 1980s [1], and more were detected in the following years [2–4]. Recently, the complete genome sequences of two isolates belonging to the species *Tombusvirus siktefluminis* (genus *Tombusvirus*) were determined [5]. Neckar River virus (NRV) was first isolated from water samples of the Neckar River near Heidelberg, Germany, by Koenig et al. in 1985 [1]. Based on serological relationships and morphological properties, this virus was identified as a novel member of the tombusvirus group and was tentatively named "tombusvirus Neckar" (TVN). In 1987, the International Committee on Taxonomy of Viruses (ICTV) included this virus in the species *Neckar River virus* of the genus *Tombusvirus*, which was renamed "*Tombusvirus neckarfluminis*" in 2023. The family *Tombusviridae* is divided into three subfamilies (*Calvusvirinae*, *Procedovirinae*, and *Regressovirinae*) and includes 18 genera and 91 species (ICTV Master Species List MSL #38). All members of this family produce icosahedral particles with diameters ranging from 30 to 35 nm, depending on the genus. Members of the genus *Tombusvirus* have a monopartite, positive single-stranded RNA genome that is approximately 4.8 kb in length. The genomic RNA of tomato busy stunt virus contains five open reading frames (ORFs) coding for five proteins: p33 and p92 (RNA-dependent RNA polymerase [RdRp]), p41 (coat protein [CP]), p22 (movement protein [MP]) and p19 (viral suppressor of RNA silencing [VSR]) [6]. An additional small putative sixth ORF located at the 3’ end of some tombusvirus genomes encodes a putative protein named pX that is 32-60 amino acids in length [7]. pX has been suggested to play a role in RNA accumulation [8]. The translation of tombusviral proteins is mediated via a 5’ cap-independent mechanism. In the 5’ UTR, several structures, including T-shaped domains and stem-loops, have been shown to be involved in replication [9, 10], and the non-polyadenylated 3’ UTR of the genomic RNA contains a 3’ cap-independent translation enhancer (3’ CITE) [11]. P33 is translated directly from the genomic RNA, and the complete RdRp (p92) is produced by readthrough of an amber stop codon [12]. The other proteins are derived from two subgenomic RNAs (sgRNAs), the larger of which, sgRNA, is about 2.1 kb in length and encodes the CP (p41), and the smaller one encodes the movement protein (p22) and silencing suppressor (p19). The p19 ORF is located within the p22 ORF, and it is generated via a leaky-scanning mechanism [13].

To date, two partial sequences of the NRV CP are available in the GenBank database (AY500887 and NC_038927), as well as a part of the NRV p33/p92 RdRp (DQ663765). In this study, the complete genome sequence of NRV (PV-0270) was determined, and this served as the basis for the successful construction of an infectious full-length clone.

## Complete genome sequencing and construction of an infectious full-length clone of NRV

**Fig. 1:**
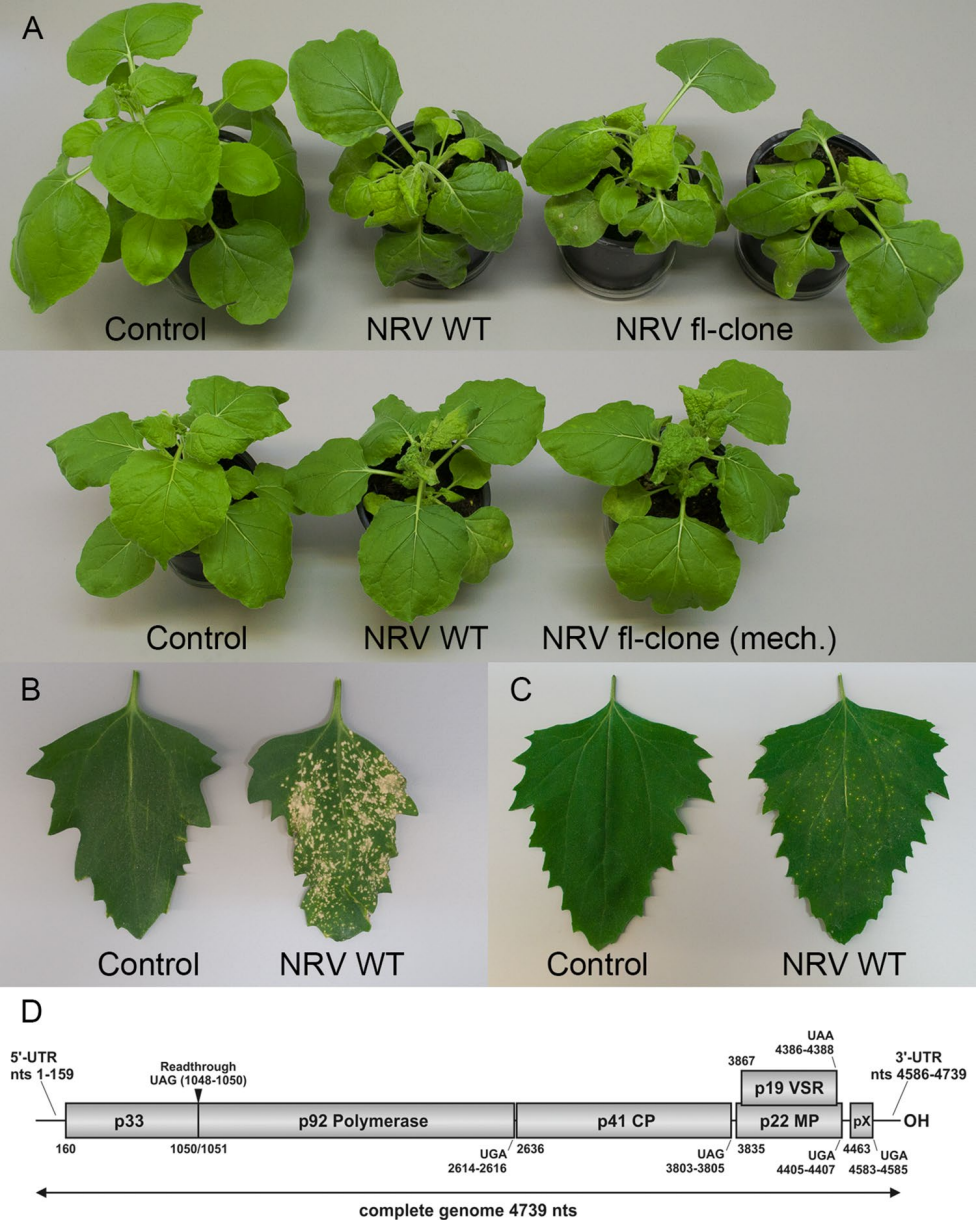
Symptoms caused by wild-type NRV and the NRV full-length clone in different plant species (**A**–**C**) and the genome organization of NRV (**D**). (**A**) Top: buffer control, an *N. benthamiana* plant that was mechanically infected with wild-type NRV, and two *N*. *benthamiana* plants that were infiltrated with the NRV full-length clone at 8 dpi. Bottom: buffer control, *N. benthamiana* plants that were mechanically infected with wild-type NRV, and the NRV full-length clone at 8 dpi. (**B**) Buffer control (left) and wild-type NRV (right) in *C*. *quinoa* at 6 dpi. (**C**) Buffer control (left) and wild-type NRV (right) in *C*. *amaranticolor* at 7 dpi. (**D**) Genome organization of NRV. UTR, untranslated region; CP, coat protein; MP, movement protein; VSR, viral suppressor of RNA silencing; pX, putative ORF with unknown function; nts, nucleotides

The German Collection of Microorganisms and Cell Cultures (DSMZ) provided a freeze-dried inoculum of an NRV-infected plant (PV-0270) that was mechanically transferred to healthy *Nicotiana benthamiana* plant. For this, approximately one-third of the freeze-dried material was added to a mixture of phosphate buffer (0.05 M KH_2_PO4, 0.05 M Na_2_HPO_4_, 1 mM EDTA, 5 mM Na-DIECA), celite, and charcoal, ground with a pestle and rubbed onto two to three mid-aged leaves of a healthy plant. Symptoms occurred 5 to 6 days postinfection (dpi) in the form of leaf curling and yellowing (Fig. 1A). A small host-range experiment was conducted in which three plants per variety were mechanically infected with NRV using symptomatic, systemically infected leaves of *N*. *benthamiana* as virus source. Two plants of each species served as buffer controls, and infection was verified by RT-PCR. Detailed results are shown in Supplementary Table S1, and the results for *Chenopodium quinoa* and *C*. *amaranticolor* are shown in Figure 1B and C. For complete genome sequencing, oligonucleotides were designed using NCBI entries DQ663765 (NRV p33/p92) and NC_038927 (NRV CP) as well as tomato bushy stunt virus sequences to generate three fragments, which were subsequently sequenced. The extreme 5’ and 3’ ends were determined by rapid amplification of cDNA ends (RACE). For the construction of an infectious full-length clone, the genome of NRV was amplified as two fragments and subsequently integrated into pDIVA (KX665539.1) via Gibson assembly. In this construct, the cloned viral cDNA is transcribed from a cauliflower mosaic virus (CaMV) 35S promoter and terminated at a hepatitis delta virus (HDV) ribozyme and a CaMV polyadenylation signal. One construct was completely sequenced and showed six nucleotide substitutions compared to the wild-type virus sequence, resulting in five amino acid changes (Supplementary Table S2). To test whether the full-length clone was infectious, *R*. *radiobacter* GV2260-mediated transformation was performed by infiltrating a bacterial suspension with an OD of 1 in inoculation buffer (10 mM MgCl_2_, 10 mM MES, 100 μM acetosyringone, pH 5.2) into the lower surface of leaves via the stomata using a needleless syringe. Symptoms were observed at approximately 5-7 dpi that resembled those of the wild-type virus symptoms but were slightly milder (Fig. 1A, top). To examine whether the slight differences in symptom expression resulted from the different methods used for infection or were due to one or more of the mutations in the NRV genome, *N. benthamiana* plants were infected using symptomatic, systemically infected plant material from a plant that had been infiltrated with the full-length NRV clone. The infection was verified by RT-PCR amplification of a 509-bp fragment from the CP cistron and subsequent sequencing. Detailed information about the methods and primers used is presented in Supplementary Table S3.

Even after using the same method (mechanical infection), the NRV full-length clone shows slightly milder symptom expression in *Nicotiana benthamiana* compared to the wild type. This might have been due to one or a combination of the mutations in the full-length clone (Supplementary Table S2). For example, the amino acid substitution in the p19 protein (VSR) might have been responsible for the milder symptoms, as p19 has been shown to influence symptom development [14, 15]. To test this, the full-length clone sequence would have to be successively adapted to the wild-type sequence and tested in subsequent infection experiments.

## Sequence comparisons and phylogenetic analysis

**Fig. 2:**
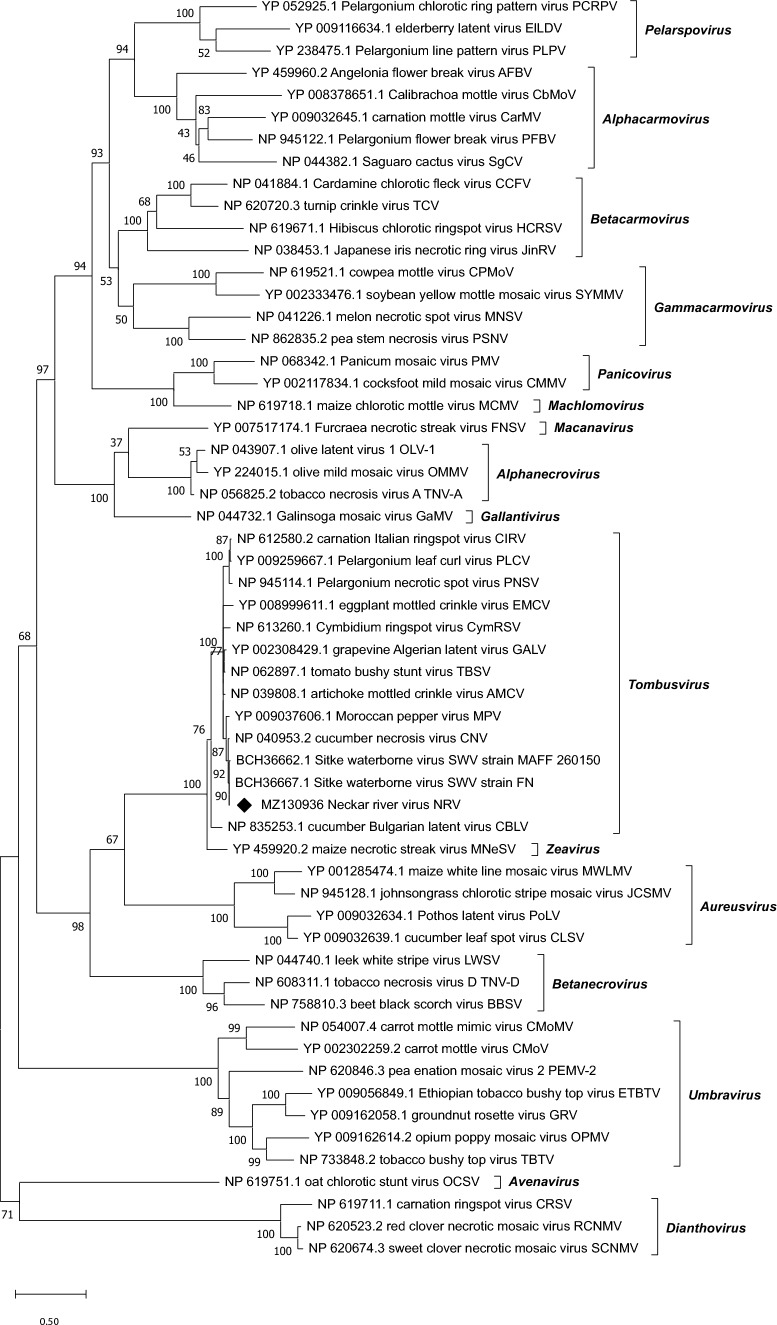
Maximum-likelihood phylogenetic tree based on 57 RdRp amino acid sequences of viruses of the family *Tombusviridae*. Number of bootstrap replications, 1000; model, LG+G+I. All positions containing gaps and missing data were eliminated (complete deletion option)

To investigate the taxonomic position of NRV within the genus *Tombusvirus* [1], a phylogenetic tree was constructed using 57 RdRp amino acid sequences from members of the family *Tombusviridae* (Fig. 2). A second, smaller phylogenetic tree based on 21 coat protein amino acid sequences of tombusviruses and dianthoviruses is shown in Supplementary Figure S1. For both trees, a MUSCLE [16] alignment was made using default parameters in MEGA X [17]. The RdRp phylogenetic tree was constructed using the LG model [18] with gamma distribution and invariant sites (G+I), and the CP phylogenetic tree was constructed using the LG model with frequencies (+F), gamma distribution, and invariant sites (G+I). One thousand bootstrap replications were performed. Gaps and missing data were eliminated. Pairwise sequence alignments to calculate percent identity values were made using the EMBOSS/Needle algorithm of the EMBL European Bioinformatics Institute (https://www.ebi.ac.uk/Tools/psa/emboss_needle/) (Supplementary Table S4).

The genome sequence of NRV is 4739 nucleotides (nt) in length, with nt 1 to 159 forming the 5’ untranslated region (UTR) and, depending on whether the putative open reading frame pX is taken into account, with either nt 4408 to 4739 or nt 4586 to 4739 forming the 3’ UTR (Fig. 1D). This genome size is comparable to that of other tombusviruses, such as tomato bushy stunt virus (TBSV, NC_001554.1, species *Tombusvirus lycopersici*), with 4776 nt. The first open reading frame (ORF1) of NRV encodes the p33 protein, and a readthrough of an amber UAG stop codon (nt 1048 to 1050) results in the expression of the complete RdRp (p92) (ORF1-RT) with a calculated molecular weight of 91.9 kDa. The CP (p41) is encoded by ORF2 and has a calculated molecular weight of 41.5 kDa. The next two open reading frames, ORF3 (p22 MP) and ORF4 (p19 VSR), overlap with each other, with p19 localized within p22. The calculated molecular weights are 21.6 kDa and 19.5 kDa, respectively. The putative ORF encoding pX could start at nt 4463 and end with a UGA stop codon at nt 4583 to 4585, from which a putative protein consisting of 40 amino acids and with a molecular weight of 4.5 kDa could be translated. Experimental evidence that this protein is expressed and has a function for the virus is still needed.

A phylogenetic tree based on RdRp sequences of tombusvirids showed that NRV clusters in the genus *Tombusvirus* as a member of the distinct species *Tombusvirus neckarfluminis*, near two members of the species *Tombusvirus siktefluminis*, cucumber necrosis virus (CNV, species *Tombusvirus cucumis*) and Moroccan pepper virus (MPV, species *Tombusvirus moroccoense*) (Fig. 2). Sequence comparisons of the CP nucleotide and amino acid sequences revealed the highest similarity to those of limonium flower distortion virus (LFDV, species *Tombusvirus limonii*), with 67.9% and 71.5% identity, respectively (Supplementary Table S4). These values clearly confirm that NRV is a member of a distinct species, as tombusviruses are considered members of the same species when the threshold of 85% CP sequence identity is exceeded. This relationship was also seen in a phylogenetic tree based on coat protein amino acid sequences of tombus- and dianthoviruses (Supplementary Fig. S1). Using the infectious full-length clone, Koch’s postulates can now be tested, and the functions of viral genes, such as the ORF encoding the putative pX protein, can be investigated experimentally.

### Supplementary Information

Below is the link to the electronic supplementary material.

Supplementary file1 (PDF 220 KB)

## Data Availability

All data supporting the findings of this study are available within the paper and its Supplementary Information.
