# Supplementary material for: The complete genome sequence of Neckar River virus confirms it to be a distinct member of the genus Tombusvirus in the family Tombusviridae
Source: Arch Virol. 2023 Nov 20;168(12):296. doi: 10.1007/s00705-023-05918-z (PMC10660902; doi:10.1007/s00705-023-05918-z)
Supplement: Supplementary file 1 — Supplementary file1 (PDF 220 KB) [file 705_2023_5918_MOESM1_ESM.pdf]

Complete genome sequence of Neckar river virus (NRV) confirms it to be a distinct member of the genus *Tombusvirus* in the family *Tombusviridae*

Thi Chi Tran[1], Edgar Maiss[1], Hanna Rose[1]

[1] Department of Phytomedicine, Institute of Horticultural Production Systems, Leibniz Universität Hannover, Herrenhäuser Str. 2, 30419 Hannover, Germany

Corresponding author:

Hanna Rose rose@ipp.uni-hannover.de

The wild type NRV sequence has been included in the NCBI database with the accession number MZ130936.

ORCiD:

Edgar Maiss 0000-0002-2035-8013

Hanna Rose 0000-0002-7226-1803

Complete genome sequencing

Verification of NRV infection was done by RT-PCR. For the latter total nucleic acids were extracted using a silica-particle based method of Menzel et al. [1]. Reverse transcription was carried out in a final volume of 20 µl. In a first step 3 µl of total nucleic acid extract and 1 µl primer CAATAGATTCCCACTCTGCCGAC (10 mM, salt free, Eurofins Genomics) were incubated at 99 °C for three minutes followed by a rapid cooling on ice. After that, 4 µl 5X RT-Buffer (Thermo Fisher Scientific), 1 µl RevertAid Reverse Transcriptase (20 U $µl^{-1}$, Thermo Fisher Scientific), 0.5 µl dNTPs (10 mM each, Thermo Fisher Scientific) and 10.5 µl H_2O were added and incubated at 42 °C for 45 min. 1 µl of the cDNA together with 7.5 µl 2X Phusion Flash High-Fidelity PCR Master Mix (Thermo Fisher Scientific), 1 µl of primers CAATAGATTCCCACTCTGCCGAC and GCTCTCGCACTCTCAAAGAAACAG (10 mM, salt free, Eurofins Genomics) each and 4.5 µl H_2O were used for the PCR reaction. The PCR program started with an initial denaturation at 98 °C for 15 s, followed by 30 cycles 98 °C for 5 s, 55 °C for 5 s and 72 °C for 15 s, finalized with 72 °C for 2 min. The amplicon covers 509 nts in the CP gene (nts 2666 – 3174). The complete genomic sequence was achieved by using the NCBI entries DQ663765 (p33/p92) and NC_038927 (CP) as templates for primer design. Subsequently, the fragment containing the gap between these two partial sequences was amplified, ligated into pTCT3 and sequenced. In a second step, a fragment located near the extreme 5'-end was amplified, ligated into pJET 1.2 blunt cloning vector via Gibson Assembly [2] and sequenced. The sense primer was derived from a tomato bushy stunt (TBSV, species *Tombusvirus lycopersici*) sequence. For the amplification of the 3' located fragment containing p22/p19, pX and parts of the 3' UTR the sense primer was derived from NC_038927 and the antisense primer from a TBSV sequence. Cloning and sequencing was performed as described before. By using rapid amplification of cDNA ends (RACE) [3], the sequences of the extreme 5'- and 3'-end were determined by direct sequencing of the PCR products. Primer sequences are shown in table S3.

Construction of an infectious full-length cDNA clone

To construct an infectious full-length cDNA clone, the genome of NRV was amplified as two fragments whereof fragment 1 contains 2315 nucleotides of the virus plus vector sequences of pDIVA (KX665539.1) as well as overlaps to fragment 2. Fragment 2 consists of 2585 nucleotides containing overlaps to fragment 1 and pDIVA. In the first step fragment 1 was cloned into the vector by Gibson Assembly, after which in the second step this construct was opened via PCR and fragment 2 was inserted as mentioned before. Primer sequences are shown in table S3. One putative full-length cDNA clone was completely sequenced and showed six nucleotide substitutions leading to five amino acid changes (Table S2). Transformation of *R.*

radiobacter GV2260 was done via electroporation and bacteria were infiltrated into *N. benthamiana* as described in [4].

Table S1: Host range of NRV

**Plant species**	**Type of infection Local (L) or systemic (S)**	**Symptom**
Chenopodium amaranticolor	L	Local lesions
Chenopodium capitatum	L	Local lesions (few)
Chenopodium quinoa	L	Local lesions
Nicotiana benthamiana	S	Yellowing, leaf curling
Nicotiana tabacum var. Xanthi nc	L	Local lesions
Nicotiana tabacum var. Samsun NN	L	Local lesions

Table S2: Nucleotide and amino acid changes between the wild type genomic sequence and the full-length cDNA clone. Nucleotide changes leading to amino acid changes are highlighted in bold.

**Nucleotide position**	**Nucleotide**	**Triplet**	**Amino acid**	**Genome feature**	**Triplet**	**Amino acid**	**Genome feature**
	WT/fl-clone	WT/fl-clone	WT/fl-clone		WT/fl-clone	WT/fl-clone	
**683**	**A/C**	**GAA/GCA**	**E/A**	**p33**			
**713**	**A/G**	**GAA/GGA**	**E/G**	**p33**			
717	A/G	CCA/CCG	P/P	p33			
**2086**	**G/T**	**GGG/UGG**	**G/W**	**p92**			
**2995**	**G/T**	**AGG/AGT**	**R/S**	**p41**			
4266	A/G	TCA/TCG	S/S	p22	**ATT/GTT**	**I/V**	**p19**

WT: wild type, **fl-clone:** full-length clone

Table S3: Oligonucleotides for the complete sequencing of NRV and construction of the infectious full-length cDNA clone.

Use		Sequence
Verification of NRV infection (fragment 505 bp)		GCTCTCGCACTCTCAAAGAAACAG<br>CAATAGATTCCCACTCTGCCGAC
Sequence primers located in the vector pTCT3 spanning the fragment.		AGGGTTTTCCCAGTCACGACGTT<br>AGGGTTTTCCCAGTCACGACGTT
Sequence primers located in the vector pJET 1.2 blunt cloning spanning the fragment.		CACCATATCCATCCGGCGTAATAC<br>CCTGATGAGGTGGTTAGCATAGTTC
Amplification of fragment containing the gap between the two database sequences (including most of p92 gene)		AAA**GGATCC**ACGCCCTCGTAGGAGACCTTATG<br>AAA**CTGCAG**CCTTGTATAGCTCGTTCCATGG
Amplification of the 5' located fragment (located within p33 gene)		GGCTCGAGTTTTTCAGCAAGATGGATAAATTGTAACTTC<br>GTAGGAGATCTTCTAGAAAGATCCACCTTAGCTCTCGCGACCTG
Amplification of the 3' located fragment (including genes p22/p19 & pX and part of 3' UTR)		GTAGGAGATCTTCTAGAAAGATGGGCTGCATTTCTGCAATGTTCCGGT<br>GGCTCGAGTTTTTCAGCAAGATCCATTGATCGTGGCGTAGTTAAGGGGC
5' RACE	reverse transcription	CCTCTGCCGTCTCTTCATAA
	PCR	GGCAGCACAATCCGTCTTCA<br>CTCAAACAGTCACGGGGGGGGGGGGGGG or ATCCTGCAGGCGCGCCCCCCCCCCCCCCCC
3' RACE	reverse transcription	ACACAATGTTACAGCTTACCCCCAGCG
	PCR	TCAAGAGGATGCCCTGAAGGCT<br>CTCAAACAGTCACGGGGGGGGGGGGGGG
Amplification of pDIVA vector		CCTCTCCAAATGAAATGAACTTCCTTATATAG<br>GGGTCGGCATGGCATCTCCACCTCCTC
Amplification of fragment 1 for Gibson Assembly into pDIVA		AGGAAGTTCATTTCATTTGGAGAGGAGAAATTCTCCAGGATTTCTCGAC<br>GAGATGCCATGCCGACCCCCACCATAATGTTGAGCGTTACTCC
Amplification of vector backbone and fragment 1 for Gibson Assembly (template: pDIVA_fragment 1)		GTGCCTGGCAGAATTCAACCTCTTC<br>GGGTCGGCATGGCATCTCCACCTCCTC
Amplification of fragment 2 for Gibson Assembly into pDIVA_fragment 1		GAAGAGGTTGAATTCTGCCAGGCAC<br>GAGATGCCATGCCGACCCGGGCTGCATTTCTGCAATGTTCCGGT

Underlined parts are vector sequences, bold parts are restriction sites.

Table S4: Percent identity values of pairwise comparisons of RdRp and CP nucleotide (nt) and amino acid (aa) sequences. **RdRp:** values exceeding 90 % are highlighted in bold, **CP:** values exceeding 60 % are highlighted in bold

			RdRp		CP	
Genus	Virus abbreviation	Accession number	nt	aa	nt	aa
Tombusvirus	AMCV	NC_001339.1	85.9	**92.4**	58.0	55.1
Tombusvirus	CIRV	NC_003500.3	77.7	79.4	54.6	50.1
Tombusvirus	CBLV	NC_004725.1	72.4	77.0	50.5	40.8
Tombusvirus	CNV	NC_001469.1	87.1	**93.0**	47.3	38.2
Tombusvirus	CymRSV	NC_003532.1	84.2	**90.0**	52.6	45.8
Tombusvirus	EMCV	NC_023339.1	84.5	**90.0**	58.7	58.4
Tombusvirus	GALV	NC_011535.1	86.4	**92.8**	50.9	46.8
Tombusvirus	HRV	NC_038690.1	na	na	49.1	36.0
Tombusvirus	LFDV	NC_038691.1	na	na	**67.9**	**71.5**
Tombusvirus	MPV	NC_020073.2	85.8	**94.0**	55.5	54.5
Tombusvirus	PLCV	NC_030452.1	76.9	79.1	57.6	55.6
Tombusvirus	PNSV	NC_005285.1	76.9	79.4	51.3	42.5
Tombusvirus	PAMV	NC_038692.1	na	na	**60.2**	57.4
Tombusvirus	SWV Lim6	NC_038693.1	na	na	50.4	41.8
Tombusvirus	SWV FN	LC564888.1	**90.7**	**94.0**	52.5	42.2
Tombusvirus	SWV MAFF 260150	LC564887.1	**91.2**	**94.1**	51.1	41.7
Tombusvirus	TBSV	NC_001554.1	87.0	**93.5**	**61.1**	58.2
Alphacarmovirus	CarMV	NC_001265.2	47.4	26.9	44.4	21.5
Alphanecrovirus	TNV-A	NC_001777.1	46.2	30.0	40.9	17.5
Aureusvirus	PoLV	NC_000939.2	49.9	36.1	53.6	44.2
Avenavirus	OCSV	NC_003633.1	45.6	28.3	44.9	26.2
Betacarmovirus	TCV	NC_003821.3	47.1	30.0	42.6	20.8
Betanecrovirus	TNV-D	NC_003487.1	47.1	35.5	37.7	15.6
Dianthovirus	CRSV	NC_003530.1	43.9	27.6	44.3	24.4
Gallantivirus	GaMV	NC_001818.1	47.6	29.0	49.0	40.0
Gammacarmovirus	MNSV	NC_001504.1	45.8	25.8	44.3	31.7
Macanavirus	FNSV	NC_020469.1	47.7	31.2	46.1	31.0
Machlomovirus	MCMV	NC_003627.1	44.2	29.0	36.5	13.1
Panicovirus	PMV	NC_002598.1	44.0	26.4	36.7	11.7
Pelarspovirus	PLPV	NC_007017.2	44.3	34.3	42.0	22.4
Umbravirus	CMoV	NC_011515.1	46.9	25.5	-	-
Zeavirus	MNeSV	NC_007729.1	66.8	70.8	40.3	15.5

na: not available

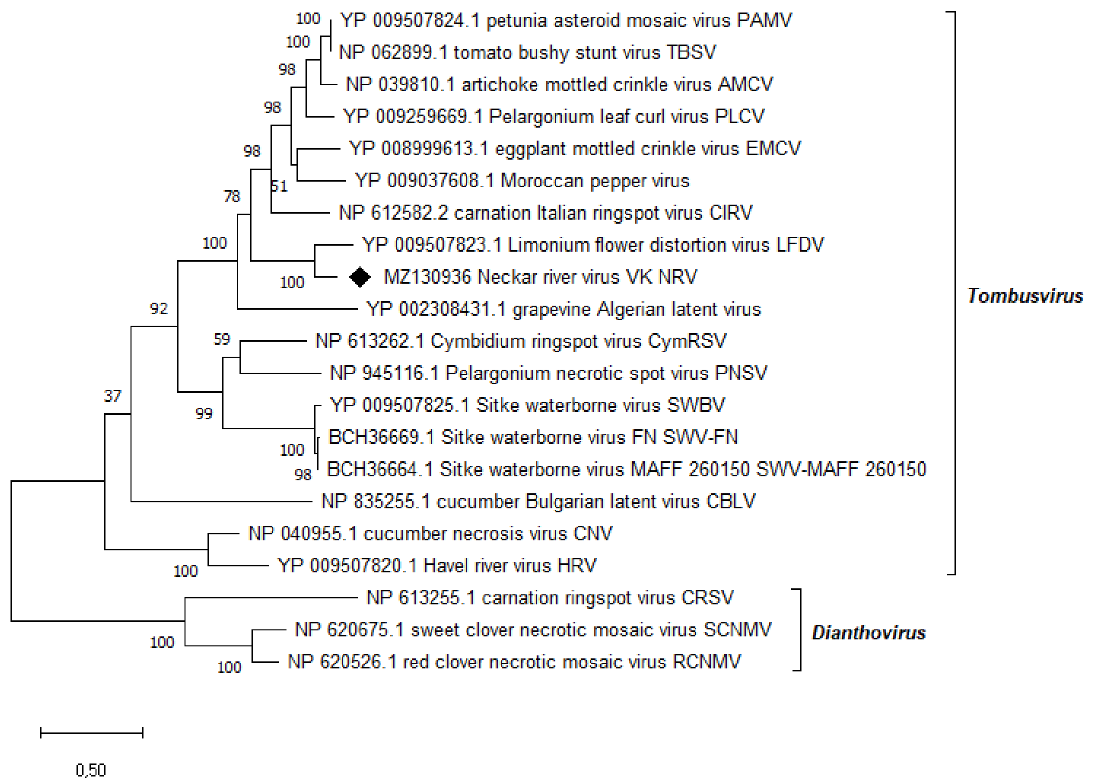

Figure S1: Maximum Likelihood phylogenetic tree based on 21 CP amino acid sequences of viruses in the genera *Tombusvirus* and *Dianthovirus*. The evolutionary history was inferred by using the Maximum Likelihood method and Le_Gascuel_2008 model [5]. The tree with the highest log likelihood (-8026.32) is shown. The percentage of trees in which the associated taxa clustered together is shown next to the branches. Initial tree(s) for the heuristic search were obtained automatically by applying Neighbor-Join and BioNJ algorithms to a matrix of pairwise distances estimated using a JTT model, and then selecting the topology with superior log likelihood value. A discrete Gamma distribution was used to model evolutionary rate differences among sites (5 categories (+G, parameter = 2.1974)). The rate variation model allowed for some sites to be evolutionarily invariable ([+I], 5.50 % sites). The tree is drawn to scale, with branch lengths measured in the number of substitutions per site. This analysis involved 21 amino acid sequences. All positions containing gaps and missing data were eliminated (complete deletion option). There were a total of 300 positions in the final dataset. Evolutionary analyses were conducted in MEGA X [6].

References

1. Menzel W, Jelkmann W, Maiss E (2002) Detection of four apple viruses by multiplex RT-PCR assays with coamplification of plant mRNA as internal control. J Virol Methods 99:81–92
2. Gibson DG, Young L, Chuang R-Y et al. (2009) Enzymatic assembly of DNA molecules up to several hundred kilobases. Nat Methods 6:343–345
3. Frohman MA, Dush MK, Martin GR (1988) Rapid production of full-length cDNAs from rare transcripts: amplification using a single gene-specific oligonucleotide primer. Proc Natl Acad Sci U S A 85:8998–9002
4. Rose H, Döring I, Vetten H-J et al. (2019) Complete genome sequence and construction of an infectious full-length cDNA clone of celery latent virus - an unusual member of a putative new genus within the *Potyviridae*. J Gen Virol 100:308–320
5. Le SQ, Gascuel O (2008) An improved general amino acid replacement matrix. Mol Biol Evol 25:1307–1320
6. Kumar S, Stecher G, Li M et al. (2018) MEGA X: Molecular evolutionary genetics analysis across computing platforms. Mol Biol Evol 35:1547–1549
